# Comparable detection of HPV using real-time PCR in paired cervical samples and concentrated first-stream urine collected with Colli-Pee device

**DOI:** 10.1016/j.diagmicrobio.2023.116160

**Published:** 2024-03

**Authors:** Pornjarim Nilyanimit, Surasith Chaithongwongwatthana, Shina Oranratanaphan, Nimesh Poudyal, Jean-Louis Excler, Julia Lynch, Sompong Vongpunsawad, Yong Poovorawan

**Affiliations:** a Center of Excellence in Clinical Virology, Department of Pediatrics, Faculty of Medicine, Chulalongkorn University, 1873 Rama 4 Road Pathumwan, Bangkok 10330, Thailand; b Department of Obstetrics and Gynecology, Faculty of Medicine, Chulalongkorn University, Thailand; c International Vaccine Institute, Seoul, South Korea

**Keywords:** HPV, Urine, Detection, Cervical swab, Cobas, Colli-Pee

## Abstract

We compared high-risk human papillomavirus (HPV) detection on first-stream urine from self-sampled collection device (Colli-Pee) and same-day clinician-collected cervical swab in 240 women. Testing with automated cobas 4800 system showed 96.7 % concordance (198 concordant-negative, 34 concordant-positive, Cohen's kappa=0.87). HPV testing on Colli-Pee urine offers advantages for acceptable non-invasive HPV screening.

Infection by the human papillomavirus (HPV) is associated with cervical cancer, a leading cause of cancer deaths in women especially those living in low- and middle-income countries [1]. Greater than 80 % of women and men are infected with HPV by the age of 45 years, with increasing risk associated with increasing number of sexual partners [2]. Infection by multiple different HPV types can occur as there are >200 HPV genotypes [3]. While low-risk HPV infection causes benign warts, high-risk HPV such as HPV16 and HPV18 is responsible for >70 % of cervical cancer [4].

Invasive clinician-collected cervical swab was considered the most reliable HPV testing procedure, but it is not universally acceptable for females of all ages and socio-cultural backgrounds. Using self-collected urine may offer an improved acceptable alternative to HPV sample collection over vaginal swab, appeal to a wider target population and enable sample collection outside of traditional healthcare settings [5,6]. Despite its preferred utility among women, the reliability and sensitivity of HPV testing in urine is questioned by some clinicians who prefer traditional methods. Furthermore, inconclusive and at times contradictory study results of HPV detected in urine reported in the literature have been insufficient to convince skeptics in accepting urine as a viable and reliable test specimen. Understandably, there is a need to develop standardized methods of collection, processing, and detection assay for urine to provide further evidence-based support for its utility. Here, we evaluated the feasibility and performance of a urine-collecting device with liquid preservative to detect high-risk HPV versus clinician-collected cervical swab.

In this prospective study, consenting healthy women who sought health check-ups at the gynecology clinic and women attending colposcopy clinic at two hospitals in Bangkok (King Chulalongkorn Memorial Hospital and Bangpakok 9 International Hospital) were consecutively enrolled from October to December 2018. Inclusion criteria were consenting healthy women 18-70 years of age who sought women health check-ups at the clinic. Specific exclusion criteria were menstruating women, expectant mothers, women who had delivery or miscarriage within the preceding six weeks, and those with urinary tract and/or cervical abnormalities. Clinic nurses previously familiar with the use of urine collection devices provided verbal instructions to the patients, who then self-collected the first-stream urine by using a Colli-Pee device (Novosanis, Wijnegem, Belgium), a plastic tube containing liquid preservative connected to a funnel with a regulator valve to volumetrically capture a total of 20 mL. Same-day clinician-collected cervical swab in Roche Cell Collection Medium (Roche Molecular System, Mannheim, Germany) was also obtained at the time of pelvic exam. In all, urine and clinician-collected cervical sample pairs from 240 women (age range 22–70 years, mean age 46 years) were tested for HPV DNA within three days. Participants provided written informed consent. The Institutional Review Board of the Faculty of Medicine of Chulalongkorn University approves this study (IRB number 362/61).

From our prior experience in handling laboratory HPV testing in urine samples, we centrifuged 10 mL of urine at 3000 rpm for 10 minutes to pellet cellular debris, and 9 mL of urine was removed. Pellet was resuspended in the remaining 1 mL urine. In parallel, cervical samples were processed according to the manufacturer's instructions (Roche). HPV detection was performed on the automated cobas 4800 system for each sample type. This assay identifies individually HPV16 and HPV18, and a grouped detection of 12 other high-risk HPV (ohrHPV) of genotypes 31, 33, 35, 39, 45, 51, 52, 56, 58, 59, 66, and 68 [7]. In this study, ohrHPV refers to these 12 genotypes, while any high-risk HPV refers to all 14 genotypes (including HPV16 and HPV18). Co-amplification of the beta-globin gene internal cellular control serves to identify false-negative results when DNA extraction and/or PCR amplification failed.

Results were categorized as concordant positive, concordant negative, or discordant. Agreement was the sum of concordant positives and negatives over total samples. Sensitivity and specificity were calculated as percentages using HPV detection in cervical samples as the comparing standard. Concordance was analyzed using Cohen's kappa statistics and were defined as slight (≤0.20), fair (0.21–0.40), moderate (0.41–0.60), substantial (0.61–0.80), and near perfect (≥0.81). Discordance was evaluated using McNemar chi-square test, and p<0.05 regarded as a statistically significant difference.

Few published studies used commercial urine collection device with proprietary preservative when testing HPV in urine. In our study, all women found self-collection of urine sample with Colli-Pee acceptable and easy. In all, HPV was detected in 16 % (38/240) of the cervical samples and in 16 % (38/240) of the urine samples (Table 1). HPV was found in 1.7 % of the samples (4/240) when tested using cervical sample but not urine, and vice versa (two-tailed McNemar p=0.72). Hence, 34 samples tested positive in both cervical and urine samples and were therefore concordant positive. There were 198 concordant negative results. Concordant positives and negatives collectively represent 96.7 % agreement between detection in urine and cervical samples (kappa=0.87).

**Table 1:** Comparison of HPV detection in 240 paired clinician-collected cervical and Colli-Pee-collected urine samples.

Method	Clinician-collected cervical samples			Sensitivity	Specificity
	Positive	Negative	Total	(95 % CI)	(95 % CI)
Urine					
Positive	34*	4	38	89 % (75 %-97 %)	98 % (95 %-99 %)
Negative	4	198	202		
Total	38	202	240		

⁎ Among the concordant positives, there were 8 HPV16, 23 ohrHPV, 2 HPV16/ohrHPV, and 1 HPV18/ohrHPV.

Sensitivity and specificity using urine compared to cervical samples for any HPV type were 89 % (95 % CI: 75 %-97 %) and 98 % (95 % CI: 95 %-99 %), respectively. Among the 34 concordant positive samples, there were 8 HPV16, 23 ohrHPV, 2 HPV16/ohrHPV, and 1 HPV18/ohrHPV. One HPV16 and 3 ohrHPV samples comprised the 4 cervical(+)/urine(-) samples. In contrast, all 4 cervical(-)/urine(+) samples were ohrHPV. When stratified by specific HPV types, the detection sensitivity and specificity for HPV16 using urine were 91 % (95 % CI: 59 %-100 %) and 100 % (95 % CI: 98 %-100 %), respectively. Furthermore, the detection sensitivity and specificity for ohrHPV were 90 % (95 % CI: 73 %-98 %) and 98 % (95 % CI: 95 %-99 %), respectively.

Early detection of precancerous HPV-infected lesions is important in preventing mortality from cervical cancer. Due to women's aversion to screening by cervical exam, a more acceptable HPV screening method for women of wider cultural and socioeconomic background is specimen self-collection. One major advantage in self-collected urine compared to self-collected vaginal swabs is its perceived ease and feasibility. In this study, urine self-collection with Colli-Pee device was well-accepted by women, provided a uniform sample volume and stability, and was supported by a prior study suggesting that the Colli-Pee-collected sample demonstrated higher HPV concentration than urine collected using a traditional cup irrespective of collection time in the morning or later in the day [8].

In our study, HPV detection in urine showed good sensitivity and specificity compared to detection in clinician-collected cervical swab. Of the 8 sample-pairs with discordant results, the absence of HPV was evenly split between cervical and urine samples. For each sample type, therefore, the false negative rate was 1.7 %. Results from our study were in agreement with previous findings using the cobas 4800 to detect HPV in urine. One study suggests that screening using urine collected in the absence of preservatives was as good as vaginal samples in identifying any high-risk HPV [9]. Our concordance in HPV detection between urine and cervical samples in this study of 96.7 % was slightly higher than one study of 88 % (125 women) and another study of 84 % (218 women) [10,11]. A Danish study also found that urine samples are non-inferior to the clinician-collected cervical samples in detecting HPV with >96 % relative sensitivity and specificity [12]. Taken together, our results were consistent with an increasing number of studies demonstrating that urine is a suitable sample type for cobas 4800 detection of HPV.

It is possible that the unique method of first-stream urine collection and processing we undertook enabled greater HPV detection than previously published studies. Combining the use of a quantitative urine collection device with a preservative solution, an added centrifugation to pellet cellular debris, and a high-throughput automated DNA extraction and detection implemented on the cobas could offer a streamlined process to detect HPV in a wider population pool, which may be useful for epidemiological surveillance and clinical implementation. Admittedly, our results may not be comparable to others for various reasons. First-stream urine as testing material may contain more exfoliated cells and contribute to better detection compared to midstream urine. Urine collected in Colli-Pee offers an equalized sample baseline as opposed to arbitrary/variable volume collection in a cup container. Finally, the urine processing method involves centrifugal concentration of cellular debris by approximately ten-fold, which may further improve detection. Taken together, urine-based testing may potentially be less expensive than standard cervical HPV screening, encourage non-attenders to screen, and may be easier to implement in resource-poor settings [13].

A major limitation of this study was the relatively small cohort. A larger cohort may yield additional detections of HPV16 and HPV18, which would increase confidence calculations. We also did not compare our HPV detection results to the histological diagnosis from biopsies, which would have been valuable. Further studies are needed in larger cohorts to evaluate cost-effectiveness and result accuracies under various testing platforms and populations.

In conclusion, the advantage of molecular testing for HPV from urine samples has the promise to be simple, accurate, and inexpensive to increase uptake in cervical cancer screening. Moreover, the highly automated and rapid HPV PCR on the cobas platform offers an opportunity to test a large population in a relatively short time and maybe ideal for epidemiological screening and vaccine effectiveness trials.

## Funding

This work was supported by The Rachadapisek Sompote Fund of Chulalongkorn University for postdoctoral fellowships to P.N., The Center of Excellence in Clinical Virology of Chulalongkorn University and Hospital (GCE 58-014-30-004), and The Bill & Melinda Gates Foundation (Grant Investment identification number INV-010411).

Author Statement not needed for Technical Notes

## CRediT authorship contribution statement

**Pornjarim Nilyanimit:** Methodology, Validation, Formal analysis, Investigation, Writing – original draft, Writing – review & editing. **Surasith Chaithongwongwatthana:** Methodology, Investigation, Writing – review & editing. **Shina Oranratanaphan:** Methodology, Investigation, Writing – review & editing. **Nimesh Poudyal:** Investigation, Project administration. **Jean-Louis Excler:** Resources, Writing – review & editing, Project administration, Funding acquisition. **Julia Lynch:** Resources, Writing – review & editing, Project administration, Funding acquisition. **Sompong Vongpunsawad:** Validation, Formal analysis, Writing – original draft, Writing – review & editing. **Yong Poovorawan:** Conceptualization, Methodology, Investigation, Resources, Writing – review & editing, Supervision, Project administration, Funding acquisition.

## Declaration of competing interest

The authors declare the following financial interests/personal relationships which may be considered as potential competing interests:

Julia Lynch reports financial support was provided by Bill and Melinda Gates Foundation. Yong Poovorawan reports financial support was provided by Chulalongkorn University. Pornjarim Nilyanimit reports financial support was provided by Rachadapisek Sompote Fund.
